# The association of healthy eating index score and n-3 fatty acid intake with cardiovascular diseases incidence and lipid biomarkers in Alberta’s tomorrow project cohort

**DOI:** 10.3389/fnut.2025.1630126

**Published:** 2025-09-24

**Authors:** Reihane Taheri, Olivia Weaver, Ming Ye, Jennifer E. Vena, Jeffrey A. Johnson, Donna Vine, Dean Eurich, Spencer D. Proctor

**Affiliations:** ^1^Metabolic and Cardiovascular Diseases Laboratory, Division of Human Nutrition, University of Alberta, Edmonton, AB, Canada; ^2^School of Public Health, University of Alberta, Edmonton, AB, Canada; ^3^Alberta’s Tomorrow Project, Cancer Care Alberta, Alberta Health Services, Calgary, AB, Canada

**Keywords:** cardiovacsular disease(s), eicosapentadienoic acid, diet, dyslipideamia, healthy eating index, n-3 fatly acids

## Abstract

**Introduction:**

Unhealthy diet and dyslipidemia are major risk factors for cardiovascular disease (CVD). Studies have shown an inverse association between greater n-3 fatty acid (FA) intake and reduced dyslipidemia and CVD risk. We aimed to assess the association of the healthy eating index (HEI) score and n-3 FA intake with CVD incidence and non-fasting RC in the Alberta’s Tomorrow Project (ATP) cohort.

**Methods:**

This is a prospective study on a subset of ATP study participants (*n* = 23,248), with the mean age of 50.2 (35-69) years, 36% male and 64% female, and no history of cancer or CVD in Alberta, Canada. Dietary intake was assessed using the Canadian Diet History Questionnaire (CDHQ), from which the Canadian HEI-2005 score and total n-3 FA intake were calculated. Lipid panel markers were measured from non-fasting blood samples, and CVD was defined using the International Statistical Classification of Diseases and Related Health Problems from linked administrative health records. The Cox proportional hazard model, linear regression, and logistic regression were used to assess the association of dietary intakes with CVD incidence, and lipid biomarkers.

**Results:**

The mean follow-up was 13.9 years. For every 1 unit increase in the HEI score, the adjusted Hazard Ratio (HR) of developing CVD decreased [HR: 0.98 (95% confidence interval (CI) 0.97–0.98), 0.99 (95%CI 0.98–0.99), and 0.97 (95%CI 0.97–0.98) in females, males, and total cohort, respectively (*p* < 0.05)]. No significant association was found between absolute n-3 FA intake (g/d) with CVD incidence. However, higher relative intake (i.e., n-3 FA as proportion of energy) increased the risk of developing CVD [HR = 1.42 (95%CI 1.1–1.84), *p* = 0.006] in males. Adjusted multivariate regression in a subset (*n* = 8,458) showed no association between n-3 FA (g/d) intake and lipid biomarkers but a significant inverse association between HEI score and non-fasting RC [coefficient: −0.006 (95%CI −0.009–−0.003) for females and −0.01 (95%CI −0.018–−0.005) for males], and TG levels [−0.01 (95%CI −0.015–−0.006) for females and −0.01 (95%CI −0.02–−0.006) for males].

**Discussion:**

Higher overall diet quality but not n-3 FA intake was associated with a lower risk of CVD incidence and non-fasting RC.

## Graphical abstract

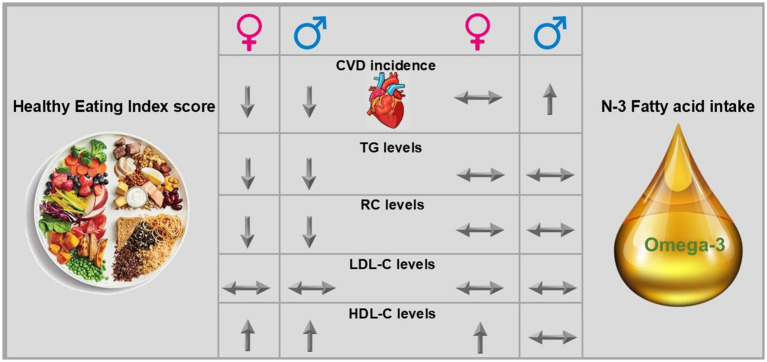

## 1 Introduction

Cardiovascular diseases (CVD) remain the leading cause of death globally, accounting for approximately 30% of all deaths (1). CVD are a group of heart and vessel disorders that present as chronic conditions, including hypertension or heart failure, as well as conditions such as stroke and heart attack (1). Risk factors for CVD are multifactorial but can include age, sex, high low-density lipoprotein cholesterol (LDL-C) and triglycerides (TG) concentration, low high-density lipoprotein cholesterol (HDL-C) concentration, dietary intake, and a high body mass index (BMI) (2–4). Atherosclerosis is the underlying etiology of most CVD. Atherosclerosis is an immuno-inflammatory process that involves the deposition of cholesterol in arteries and aggregation of immune cells and smooth muscle cells, resulting in the formation of plaque, stenosis, and reduced blood flow to organs (5–9). Dyslipidemia, defined as high LDL-C, TG, or low HDL-C, is associated with higher cholesterol deposition in arteries and is a major risk factor for atherosclerosis (10). However, other classes of lipoproteins in the non-fasting state, including chylomicron remnants, very low-density lipoproteins (VLDL), and intermediate-density lipoproteins (IDL), have also been shown to contribute to the cholesterol deposition in arteries and progression of atherosclerosis (8). In order to consider the cholesterol content of these particles, remnant cholesterol (RC) has been introduced, which estimates the cholesterol content of chylomicron remnants, VLDL, and IDL in either the fasting or the non-fasting state (11). Importantly, recent studies have shown that non-fasting remnant cholesterol (RC) is an independent causal risk factor for CVD (12–14). Previous results from the Alberta’s Tomorrow Project (ATP) cohort (*n* = 13,988) showed that with each 1 mmol/L increase in non-fasting RC level, the likelihood of CVD incidence increased by 48%. Further, results in the ATP cohort showed that those with CVD incidence (vs. without) had a significantly higher mean RC (but not LDL-C) (15).

An unhealthy diet remains a significant modifiable risk factor for CVD incidence. A healthy diet is recommended for both the prevention and management of CVD by Canadian and American guidelines (16–18). The American Heart Association guideline to improve cardiovascular health states that fatty acids (FA) from diet can affect lipoprotein levels and recommends substituting saturated FA with n-3 and n-6 poly-unsaturated FA (PUFA) wherever possible (19). Most epidemiological and clinical trial studies indicate that higher n-3 PUFA intake is associated with lower risk of CVD especially in those with existing coronary heart disease (20, 21). N-3 PUFA exert their protective effect through reduction of inflammation and TG levels (22). Some smaller randomized controlled trials (RCTs) have demonstrated that n-3 PUFAs can reduce levels of both TG and RC, but not necessarily LDL-C (23–25). Other larger RCTs show that n-3 PUFAs [specifically eicosapentaenoic acid (EPA)] can improve CVD outcomes and lower TG but have no consistent effect on other lipoproteins (26, 27). Some review studies suggest that because n-6 and n-3 PUFAs are precursors of pro-inflammatory and anti-inflammatory cytokines, respectively, and they compete for the same enzyme, higher intake of n-6 PUFA may increase low-grade inflammation, oxidative stress, oxidized LDL-C, and ultimately the risk of CVD (19, 28). Evidence from human studies does not align with this theory and most cohort studies showed that in fact there is an inverse association between n-6 PUFA intake and CVD, especially when the n-6 PUFA is substituting saturated fatty acids (28–30).

In the current study, we aimed to assess the association of a diet quality [using healthy eating index (HEI) score] and n-3 FA intake with risk of future CVD incidence and non-fasting RC, TG, LDL-C, and HDL-C levels in Albertans from the ATP cohort in Canada.

## 2 Methods and materials

### 2.1 Participants

This study was approved by the University of Alberta Human Research Ethics Committee (Pro00073641) to obtain corresponding health data from Alberta Health administrative health record registries. Additional details of the methodology have been described elsewhere (31). Briefly, from 2000–2015, ATP recruited almost 55,000 adults aged 35–69 years who had no history of cancer other than non-melanoma skin cancer, could complete questionnaires in English, and planned to reside in Alberta, Canada, for at least 1 year. This study includes participants who were recruited from 2000–2008 and completed Health and Lifestyle Questionnaires (HLQ) as well as diet and physical activity questionnaires (described below) in that same time period, who also consented to linkage to administrative health records, and subsequently provided a blood sample in 2009–2015. The demographic characteristics of participants have been described previously (15, 31).

**Figure 1:**
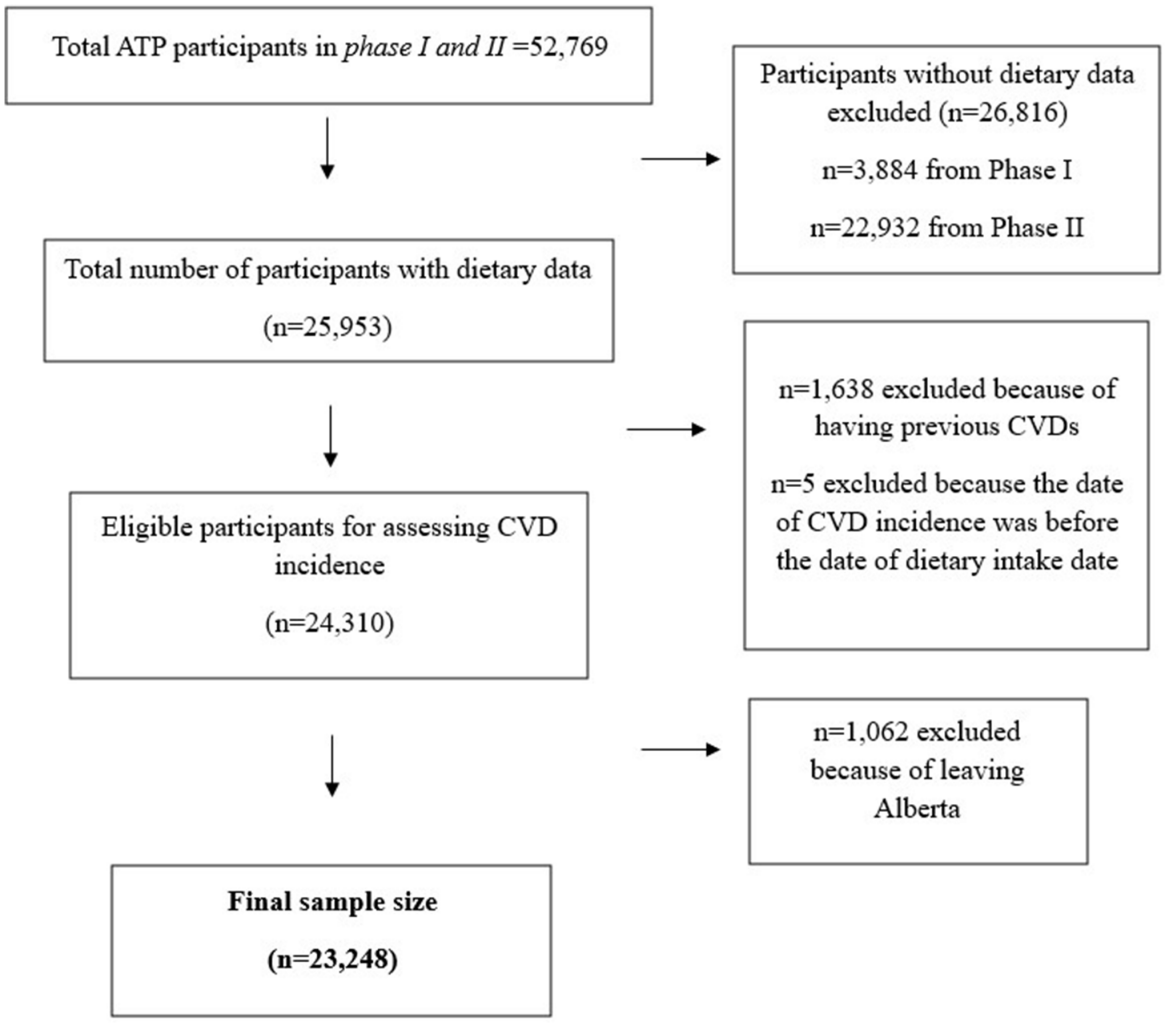
Flowchart of sample selection.

Participants (*n* = 29,878) were originally recruited using random digit dialing sample selection and were mostly female (64%), Caucasian (91%), living in urban areas (77%), and had an average age of 50 ± 9 years (31). For this study, we further selected participants who did not have prevalent CVD at the time of enrollment. The main exclusion criteria included leaving Alberta, because the incidence of CVD was ascertained using linked provincial administrative health records (described below). Our final sample size was *n* = 23,248 participants; Figure 1 shows the flowchart of sample selection.

### 2.2 Dietary intake and physical activity

At the time of recruitment to ATP study (during 2000–2008), following self-administered ATP questionnaires were mailed to participants who consented to participate in ATP study: HLQ, for obtaining personal and family health history, reproductive health, and demographic characteristics, Canadian Diet History Questionnaire I (CDHQ-I) to obtain past 12 months dietary intake, and Past-Year Total Physical Activity Questionnaire (PYTPAQ) to obtain past 12 months physical activity. The PYTPAQ assesses the type, duration, frequency, and intensity of activities during the past year (31). The reliability and validity of PYTPAQ in the Canadian population have been assessed previously (32). CDHQ-I is a 124-item food frequency questionnaire modified from the DHQ from the U. S. National Cancer Institute, with 145 questions about food items, beverages, and dietary supplements intake. Details of the CDHQ and the validity of the questionnaire for use in Canadian population have been previously published (33).

CDHQ-I data was analyzed using Diet*Calc (version 1.4.2, National Cancer Institute, Bethesda, MD, USA) to assess the mean daily intake of energy, macronutrients, and micronutrients. To assess the n-3 FA intake, we summated the value of alpha linolenic acid [ALA (C18:3n3)], EPA (C20:5n3), docosahexaenoic acid [DHA(C22:6n3)], and docosapentaenoic acid [DPA(C22:5n3)]. For assessing diet quality, the American 2005 HEI adapted for Canada was used (34). The details of HEI score and the validity to use in Canadian population have been previously published (34). The HEI score ranges between 0–100, with higher scores indicating higher diet quality via adherence to dietary recommendations. It accounts for two primary aspects of diet quality, which are adequacy (adequate intake of healthy foods and nutrients) and moderation (moderate intake of unhealthy foods and nutrients). HEI scores <50 represent poor diet quality, 50–80 represent moderate diet quality that needs improvement, and higher than 80 represent high diet quality (34).

### 2.3 Health information, comorbidities and cardiovascular diseases

The information for CVD and comorbidity prevalence and/or incidence were obtained via data linkage to Alberta Health registry databases (including emergency department, physician claims, and Discharge Abstract Database) using the personal health numbers provided by participants (31).

Cases of CVD were identified using codes from the International Statistical Classification of Diseases and Related Health Problems (ICD) (31). Specifically, in physician claim data, ICD-9 is used all the time, whereas in inpatient (hospitalization) data, ICD-9 was used before 2003, and after 2003, ICD-10 was implemented. Therefore, in ATP cohort both ICD-9 and 10 have been used. Procedures were identified with the Canadian Classification of Diagnostic, Therapeutic, and Surgical Procedures codes, following the definitions provided by the Alberta Diabetes Surveillance System (15). The primary outcome of this study was the CVD composite incidence, which is a cluster of CVD or procedures including ischemic heart disease (IHD), myocardial infarction (MI), angina, heart failure (HF), transient ischemic attack (TIA), acute ischemic stroke (AIS), percutaneous coronary intervention (PCI) and coronary artery bypass graft (CABG) defined by Clair et al. (35) in those without previous CVD, before or within 6 months of enrolment to ATP or within 1 year of data linkage to the Alberta Health databases. The Elixhauser index score (score ranges 0–30) was generated previously as a continuous variable (with Quan coding algorithm) (36) to indicate the presence of 30 different comorbidities that do not overlap with CVD (15, 37).

The menopause status for females was obtained via self-reported HLQ and clinical outcomes in routine evaluation (CORE) questionnaires.

### 2.4 Lipid biomarkers

In 2008, ATP joined the Canadian Partnership for Tomorrow’s Health (CanPath). As part of joining CanPath, ATP invited existing participants to complete further questionnaires and attend a study center to provide blood and urine samples. Non-fasting blood (~50 mL) sample was drawn at one time point, aliquoted to serum and plasma, and stored at −80°C for further analysis, with the majority of samples frozen within 2 h of blood draw (31). In 2017–2020 participants’ serum (0.5 mL) was used for assessing lipid biomarkers. HDL-C, TG, and total cholesterol (TC) were measured directly by Calgary Laboratory Services, an authorized clinical laboratory in Alberta (31). Non-HDL-C, RC, and LDL-C (by Friedewald formula) were calculated by the following equations:


Non−HDL−C=TC−HDL−C

RC=TC−(LDL−C+HDL−C)

LDL−C=TC−HDL−C−TG/5

Of the participants included in this study, a subset (*n* = 8,747) had lipid biomarkers data available.

### 2.5 Statistical analysis

Data was analyzed using STATA SE version 16.1. Descriptive statistics are shown as mean±standard deviation (SD) for continuous variables and percentage (number) for categorical variables. To compare the means at baseline, an independent t-test was used. Logistic regression was used for assessing the odds of occurring binomial outcome variable (CVD incidence), Cox proportional hazard model was used for assessing the relative risk of binomial outcome variable (CVD incidence), linear regression was used for assessing the linear relationship between exposure variables and continuous outcome variables (lipid biomarkers). Unadjusted and adjusted logistic regression [odds ratio (OR) and 95% confidence interval (95%CI)] was used for assessing the association of n-3 FA intake and HEI score with CVD incidence. The adjusted Cox proportional hazard model was used to calculate the hazard ratio (HR) and 95% CI. The adjusted linear regression [Coefficient (coef) and 95%CI] was used for the association of HEI score and n-3 FA intake with lipid biomarkers. The models were stratified by sex. For adjusting the confounders, we used two models; Model a: age, BMI (kg/m^2^), total energy(kcal), carbohydrate (%), protein (%), and fat (%) intake, Elixhauser index, as well as HEI score were adjusted to assess the association of n-3 FA intake (g/d), energy from n-3 FA (%), EPA intake (g/d), and energy from EPA (%) with CVD incidence and lipid biomarkers. Model b: age, BMI (kg/m^2^), total energy(kcal), carbohydrate (%), protein (%), and fat (%) intake, and Elixhauser index and were adjusted to assess the association of HEI score with CVD incidence and lipid biomarkers. *p*-value<0.05 (alpha error 5%) was considered as significant difference.

## 3 Results

The proportion of CVD incidence in our ATP cohort sample (*n* = 23,248) with a mean follow-up of 13.91 ± 4.70 years was 24.2%. Out of 23,248 participants, 4 participants had good diet quality (HEI score>80); 65.9% had moderate diet quality (HEI score of 51–80), and 34.1% had poor diet quality (HEI score of ≤50). The mean of HEI score in good, moderate, and poor diet quality groups were 81.15 ± 1.26, 59.00 ± 5.94, and 43.08 ± 5.40, respectively. Those in the moderate HEI score category had significantly lower CVD incidence compared to low HEI scores (22% vs. 26%, Chi square *p*-value<0.001).

### 3.1 Characteristics of the participants stratified by CVD incidence

Table 1 shows the characteristics of participants with and without incident CVD. Those with CVD incidence (compared to those without) were significantly older (54 ± 9 vs. 49 ± 8.7 years, *p*-v < 0.001), had higher BMI (29.8 ± 6 vs. 28.2 ± 5.6 kg/m^2^, *p*-v < 0.001), slightly lower HEI score (52.6 ± 9.6 vs. 53.8 ± 9.4, *p*-v < 0.001), and higher Elixhauser score means (2.1 ± 1.9 vs. 1.6 ± 1.6, *p*-v < 0.001). Comparing the baseline characteristics in participants with vs. without CVD incidence, no significant difference was seen in the means of total n-3 FA intake (g/d) (1.43 ± 0.75 vs. 1.43 ± 0.73), n-3 FA as proportion of total energy (%) (0.70 ± 0.21 for both), EPA intake (g/d) (0.02 ± 0.03 for both), EPA as proportion of total energy (%) (0.01 ± 0.01 for both), n-3 to n-6 PUFA ratio (0.11 ± 0.02 for both), and physical activity level (1.91 ± 0.42 vs. 1.98 ± 7.54).

**Table 1:** The characteristics of participants with and without CVD.

Variables	Total participants (*N* = 23,248)	without CVD (*N* = 17,612)	With CVD (*N* = 5,636)	*p*-v (with vs. without CVD)
Age at baseline (years)	50.27 ± 9.06	49.07 ± 8.73	54.04 ± 9.04	<0.001*
BMI (kg/m^2^)	28.60 ± 5.74	28.20 ± 5.60	29.85 ± 5.98	<0.001*
Total energy intake (kcal)	1867.43 ± 865.95	1867.32 ± 858.63	1867.79 ± 888.51	0.9
Energy from carbohydrates (%)	50.36 ± 8.52	50.28 ± 8.39	50.58 ± 8.91	0.02*
Energy from fat (%)	32.75 ± 6.86	32.79 ± 6.81	32.64 ± 7.04	0.1
Energy from protein (%)	15.97 ± 2.95	16.02 ± 2.92	15.82 ± 3.02	<0.001*
Energy from PUFA (%)	7.05 ± 2.02	7.05 ± 2.02	7.04 ± 2.04	0.7
Energy from SFA (%)	10.75 ± 2.79	10.77 ± 2.77	10.67 ± 2.87	0.02*
Energy from MUFA (%)	12.37 ± 2.94	12.38 ± 2.92	12.33 ± 2.98	0.3
Energy from N-3 FA (%)	0.70 ± 0.21	0.70 ± 0.21	0.70 ± 0.21	0.9
N-3 FA intake (g/d)	1.43 ± 0.0.73	1.43 ± 0.73	1.43 ± 0.75	0.9
N-3/N-6 PUFA ratio	0.11 ± 0.02	0.11 ± 0.02	0.11 ± 0.02	0.6
N-3/total fat ratio	0.02 ± 0.00	0.02 ± 0.00	0.02 ± 0.00	0.08
EPA intake (g/d)	0.02 ± 0.03	0.02 ± 0.03	0.02 ± 0.03	0.6
Energy from EPA (%)	0.01 ± 0.01	0.01 ± 0.01	0.01 ± 0.01	0.7
HEI score	53.58 ± 9.50	53.87 ± 9.42	52.67 ± 9.67	<0.001*
Physical activity level	1.96 ± 6.56	1.98 ± 7.54	1.91 ± 0.42	0.23
Elixhauser score	1.76 ± 1.72	1.64 ± 1.64	2.14 ± 1.88	<0.001*

SFA, saturated fatty acid; MUFA, mono-unsaturated fatty acid. Continuous variables are reported as mean ± SD, and categorical variables are reported as proportion (percentage). An independent two sample t-test was used to compare the means between groups. *Significant *p*-values (<0.05) are indicated with asterisk. The energy of macro/micronutrients (%) is calculated as the mean energy of the specific macro/micronutrient (kcal) divided by the mean total energy intake multiplied by 100.

### 3.2 Characteristics of the participants stratified by sex

Table 2 shows the characteristics of male and female participants. The incidence of CVD in females (*n* = 14,729) in comparison to males (*n* = 8,519) was significantly lower (21.93 vs. 28.24%, *p*-v < 0.001). The mean intake of n-3 FA (g/d) and EPA (g/d) in females compared to males were also significantly lower (1.3 ± 0.6 vs. 1.6 ± 0.8, and 0.024 ± 0.029 vs. 0.029 ± 0.034 p = v < 0.001). The ratio of n-3 FA to total energy and EPA to total energy (%) was slightly higher in females vs. males (0.7 ± 0.2 vs. 0.6 ± 0.2, and 0.013 ± 0.015 vs. 0.012 ± 0.013, *p*-v < 0.001). The mean HEI and Elixhauser scores were significantly higher in females (55.2 ± 9.4 vs. 50.7 ± 8.8, and 1.9 ± 1.8 vs. 1.4 ± 1.5, *p*-v < 0.001).

**Table 2:** The characteristics of participants in females and males.

Variables		Males (*N* = 8,519)	Females (*N* = 14,729)	*p*-v
Age at baseline (years)		50.16 ± 8.98	50.34 ± 9.11	0.15
CVD incidence [number (%])	Yes	2,406 (28.24%)	3,230 (21.93%)	<0.001*
	No	6,113 (71.76%)	11,499 (78.07%)	
BMI (kg/m^2^)		29.06 ± 4.60	28.33 ± 6.22	<0.001*
Total Energy intake (kcal)		2257.17 ± 1018.72	1642.01 ± 666.87	<0.001*
Energy from carbohydrates (%)		48.62 ± 8.37	51.36 ± 8.44	<0.001*
Energy from fat (%)		33.17 ± 6.77	32.51 ± 6.91	<0.001*
Energy from protein (%)		15.69 ± 2.95	16.13 ± 2.95	<0.001*
Energy from PUFA (%)		6.75 ± 1.85	7.23 ± 2.10	<0.001*
Energy from SFA (%)		11.03 ± 2.76	10.58 ± 2.80	<0.001*
Energy from MUFA (%)		12.76 ± 2.93	12.14 ± 2.91	<0.001*
Energy from n-3 FA (%)		0.65 ± 0.19	0.73 ± 0.022	<0.001*
N-3 FA intake (g/d)		1.62 ± 0.81	1.32 ± 0.66	<0.001*
EPA intake (g/d)		0.03 ± 0.03	0.02 ± 0.03	0 < 001*
Energy from EPA (%)		0.01 ± 0.01	0.01 ± 0.01	<0.001*
HEI score		50.78 ± 8.85	55.20 ± 9.49	<0.001*
Physical activity level		2.07 ± 10.84	1.90 ± 0.38	0.14
Elixhauser score		1.43 ± 1.52	1.95 ± 1.79	<0.001*

Continuous variables are reported as mean±SD, and categorical variables are reported as number (percentage). CVD incidence was compared between groups using the Fisher’s exact test. An independent sample *t*-test was used to compare the means between groups. *Significant *p*-values (<0.05) are indicated with asterisk. The energy of macro/micronutrients (%) is calculated as the mean energy of the specific macro/micronutrient (kcal) divided by mean total energy intake multiplied by 100.

Table 3 shows the characteristics of participants with and without CVD stratified by sex. In both sexes, those with CVD incidence were older and had higher BMI, lower HEI score, and higher Elixhauser score (*p*-v < 0.001). In females (with vs. without CVD), there was no significant difference in either n-3 FA intake (g/d) or n-3 FA as proportion of total energy (%). In males with CVD (versus without CVD), the mean of n-3 FA intake (g/d) was slightly lower (1.6 ± 0.8 vs. 1.64 ± 0.8, *p*-v = 0.03) but, the n-3 FA as proportion of total energy (%) was slightly higher (0.66 ± 0.2 vs. 0.65 ± 0.2, *p*-v = 0.006).

**Table 3:** The characteristics of participants with and without CVD stratified by sex.

Variables		Without CVD (*N* = 17,612)	With CVD (*N* = 5,636)	Difference	*p*-v
Age at baseline (years)	Female (64%)	49.20 ± 8.78	54.9 ± 9.09	5.2	<0.001*
	Male (36%)	48.82 ± 8.63	53.57 ± 8.95	4.7	<0.001*
BMI (kg/m^2^)	Female (64%)	27.93 ± 6.12	29.76 ± 6.68	1.8	<0.001*
	Male (36%)	28.70 ± 4.42	29.97 ± 4.90	1.2	<0.001*
Total Energy intake (kcal)	Female (64%)	1646.04 ± 657.21	1627.66 ± 700.10	−18.3	0.1
	Male (36%)	2283.55 ± 1022.91	2190.16 ± 1005.10	−93.4	<0.001*
Energy from carbohydrates (%)	Female (64%)	51.14 ± 8.29	52.14 ± 8.92	1	<0.001*
	Male (36%)	48.67 ± 8.34	48.49 ± 8.45	−0.2	0.3
Energy from fat (%)	Female (64%)	32.58 ± 6.82	32.26 ± 7.19	−0.3	0.02*
	Male (36%)	33.18 ± 6.76	33.14 ± 6.81	−0.04	0.8
Energy from protein (%)	Female (64%)	16.20 ± 2.92	15.86 ± 3.04	−0.3	<0.001*
	Male (36%)	15.66 ± 2.90	15.76 ± 3.00	0.1	0.1
Energy from PUFA (%)	Female (64%)	7.22 ± 2.09	7.23 ± 2.12	0	0.8
	Male (36%)	6.73 ± 1.84	6.79 ± 1.90	0.06	0.2
Energy from SFA (%)	Female (64%)	10.61 ± 2.77	10.45 ± 2.91	−0.16	0.003*
	Male (36%)	11.06 ± 2.76	10.97 ± 2.79	−0.09	0.2
Energy from MUFA (%)	Female (64%)	12.17 ± 2.89	12.02 ± 3.01	−0.1	0.01*
	Male (36%)	12.77 ± 2.95	12.75 ± 2.88	0	0.7
Energy from N-3 FA (%)	Female (64%)	0.73 ± 0.22	0.73 ± 0.21	0	0.8
	Male (36%)	0.65 ± 0.19	0.66 ± 0.20	0.01	0.006*
N-3 FA intake (g/d)	Female (64%)	1.33 ± 0.66	1.32 ± 0.68	−0.01	0.3
	Male (36%)	1.64 ± 0.81	1.59 ± 0.81	−0.04	0.03*
N-3/N-6 PUFA ratio	Female (64%)	0.11 ± 0.02	0.11 ± 0.02	0	0.7
	Male (36%)	0.11 ± 0.02	0.11 ± 0.02	0	0.06
N-3/total fat ratio	Female (64%)	0.02 ± 0.00	0.02 ± 0.00	0.0003	0.002*
	Male (36%)	0.02 ± 0.00	0.02 ± 0.00	0.0004	0.001*
EPA intake (g/d)	Female (64%)	0.02 ± 0.03	0.02 ± 0.03	0	0.2
	Male (36%)	0.03 ± 0.03	0.03 ± 0.03	0	0.5
Energy from EPA (%)	Female (64%)	0.01 ± 0.01	0.01 ± 0.01	0	0.6
	Male (36%)	0.01 ± 0.01	0.01 ± 0.01	0	0.4
HEI score	Female (64%)	55.43 ± 9.35	54.39 ± 9.93	−1	<0.001*
	Male (36%)	50.95 ± 8.86	50.36 ± 8.79	−0.6	0.005*
Physical activity level	Female (64%)	1.90 ± 0.37	1.89 ± 0.41	0	0.5
	Male (36%)	2.12 ± 12.79	1.93 ± 0.44	−0.2	0.2
Elixhauser score	Female (64%)	1.83 ± 1.72	2.38 ± 1.96	0.5	<0.001*
	Male (36%)	1.27 ± 1.41	1.82 ± 1.72	0.5	<0.001*

Total number of females = 14,729 (11,499 without CVD, 3,230 with CVD), Total number of males = 8,519 (6,113 without CVD, 2,406 with CVD). Continuous variables are reported as mean ± SD. An independent sample t-test was used to compare the means between groups.*Significant *p*-values (<0.05) are indicated with asterisk. The energy of macro/micronutrients (%) is calculated as the mean energy of the specific macro/micronutrient (kcal) divided by mean total energy intake multiplied by 100.

### 3.3 Association of n-3 FA intake and HEI score with CVD incidence

The unadjusted and adjusted logistic regression model was used to assess the association of HEI score and n-3 FA intake with CVD incidence (Table 4). After adjusting for confounders (age, BMI, total energy, carbohydrate, fat, and protein intake (%), and Elixhauser score), in either women, men, or the combined total cohort, higher HEI score was associated with lower odds of developing CVD [OR: 0.97 (95%CI 0.97–0.98), *p*-v < 0.001, OR: 0.98 (95%CI 0.98–0.99), *p*-v = 0.01, and OR: 0.97 (95%CI 0.97–0.97) *p*-v < 0.001 respectively]. After adjusting for the confounders (age, BMI, total energy, carbohydrate, fat, and protein intake (%), Elixhauser score, and HEI score) for assessing the association of n-3 FA intake (g/d) with CVD incidence, no association was found in women. However, for males and the total cohort, higher n-3 FA intake (g/d) was associated with higher odds of CVD incidence [OR: 1.13 (95%CI 1.01–1.27), *p*-v = 0.03, and OR: 1.09 (95%CI 1.00–1.18), *p*-v = 0.02, respectively]. Higher N-3 FA to total energy ratio (%) was associated with a higher odds of CVD incidence in males [OR: 1.62 (95%CI 1.19–2.21), *p*-v = 0.002], and no association was found in females and the total cohort.

**Table 4:** Logistic regression for assessing the relation of dietary variables with CVD incidence odds.

Variable		OR (95%CI)	*p*-v	Sex	Adjusted OR (95%CI)	*p*-v
Energy from N-3 FA (%)^a^	Female (14,729)	1.02 (0.87–1.21)	0.8	Female (14,690)	1.06 (0.84–1.34)	0.5
	Male (8,519)	1.39 (1.1–1.76)	0.006^a*^	Male (8,498)	1.62 (1.19–2.21)	0.002*
	Total (23,248)	1.005 (0.87–1.15)	0.9	Total (23,188)	1.13 (0.94–1.37)	0.1
N-3 FA intake (g/d)^a^	Female (14,729)	0.97 (0.91–1.03)	0.3^a*^	Female (14,690)	1.04 (0.92–1.17)	0.4
	Male (8,519)	0.93 (0.88–0.99)	0.03	Male (8,498)	1.13 (1.01–1.27)	0.03*
	Total (23,428)	1.00 (0.96–1.04)	0.9	Total (23,188)	1.09 (1.0–1.18)	0.02*
N-3/N-6 PUFA ratio^a^	Female (14,729)	1.27 (0.28–5.71)	0.7	Female (14,690)	0.44 (0.08–2.26)	0.3
	Male (8,519)	5.87 (0.95–36.33)	0.57	Male (8,498)	4.3 (0.61–30.44)	0.1
	Total (23,248)	1.35 (0.42–4.31)	0.6	Total (23,188)	0.76 (0.21–2.67)	0.6
HEI score^b^	Female (14,729)	0.98 (0.98–0.99)	<0.001^a*^	Female (14,690)	0.97 (0.97–0.98)	<0.001*
	Male (8,519)	0.99 (0.98–0.99)	0.006^a*^	Male (8,498)	0.98 (0.98–0.99)	0.01*
	Total (23,248)	0.98 (0.98–0.98)	<0.001^a*^	Total (23,188)	0.97 (0.97–0.97)	<0.001*
EPA intake (g/d)^a^	Female (14,729)	0.46 (0.11–1.78)	0.26	Female (14,690)	1.1 (0.23–5.21)	0.9
	Male (8,519)	0.61 (0.15–2.5)	0.49	Male (8,498)	0.65 (0.13–3.23)	0.6
	Total (23,248)	0.81 (0.31–2.13)	0.67	Total (23,188)	1.1 (0.36–3.3)	0.8
Energy from EPA (%)^a^	Female (14,729)	0.59 (0.04–7.25)	0.7	Female (14,690)	1.7 (0.1–27.93)	0.6
	Male (8,519)	3.8 (0.12–115.97)	0.4	Male (8,498)	0.27 (0.006–11.7)	0.5
	Total (23,248)	0.68 (0.09–5.13)	0.7	Total (23,188)	0.76 (0.08–7.04)	0.8

The crude and adjusted logistic regressions were used to calculate the odds ratio and 95% confidence intervals. *Significant *p*-values (<0.05) are indicated with asterisk. a: Adjusted model a. b: Adjusted model b.

Table 5 shows the adjusted and unadjusted HR for the association of HEI score, n-3 FA intake (g/d), and n-3 FA to total energy ratio (%) with CVD incidence. After adjusting for confounders (model a), no association was found between n-3 FA (g/d) and CVD incidence in women, men, and the total cohort. Higher n-3 FA as proportion of total energy (%) was associated with increased CVD risk in males (HR: 1.42, 95% CI 1.1–1.84, *p*-v = 0.006). After adjusting for confounders (model b) higher HEI score was associated with reduced risk of CVD incidence in women, men, and the total cohort (HR: 0.98, 95%CI 0.97–0.98, *p*-v < 0.001, HR: 0.99, 95%CI 0.98–0.99, *p*-v = 0.004, HR: 0.97, 95%CI 0.97–0.98, *p*-v < 0.001, respectively). No significant association was found between EPA (g/d) and CVD incidence in all groups.

**Table 5:** Hazard ratio for assessing the relation of dietary variables with CVD incidence risk.

Variable		HR (95%CI)	*p*-v	Sex	Adjusted HR (95%CI)	*p*-v
Energy from N-3 FA (%)^a^	Female (14,729)	1.03 (0.88–1.2)	0.69	Female (14,690)	1.00 (0.82–1.22)	0.96
	Male (8,519)	1.32 (1.08–1.61)	0.005*	Male (8,498)	1.42 (1.1–1.84)	0.006*
	Total (23,248)	1.01 (0.89–1.14)	0.85	Total (23,188)	1.04 (0.89–1.22)	0.57
intake-3 FA intake (g/d)^a^	Female (14,729)	0.97 (0.92–1.02)	0.28	Female (14,690)	1.09 (0.91–1.11)	0.87
	Male (8,519)	0.93 (0.89–0.97)	0.018*	Male (8,498)	1.09 (0.99–1.2)	0.054
	Total (23,428)	0.99 (0.96–1.03)	0.78	Total (23,188)	1.05 (098–1.12)	0.11
N-3/N-6 PUFA ratio^a^	Female (14,729)	1.05 (0.27–3.9)	0.93	Female (14,690)	0.4 (0.1–1.6)	0.19
	Male (8,519)	4.4 (0.93–20.68)	0.06	Male (8,498)	3.52 (0.69–17.8)	0.12
	Total (23,248)	1.16 (0.42–3.21)	0.76	Total (23,188)	0.63 (0.22–1.81)	0.39
HEI score^b^	Female (14,729)	0.98 (0.98–0.99)	<0.001*	Female (14,690)	0.98 (0.97–0.98)	<0.001*
	Male (8,519)	0.99 (0.98–0.99)	0.003*	Male (8,498)	0.99 (0.98–0.99)	0.004*
	Total (23,248)	0.98(0.98–0.99)	<0.001*	Total (23,188)	0.97 (0.97–0.98)	<0.001*
EPA intake (g/d)^a^	Female (14,729)	0.69 (0.2–2.2)	0.54	Female (14,690)	1.11 (0.3–4.15)	0.86
	Male (8,519)	0.75 (0.22–2.46)	0.64	Male (8,498)	0.62 (0.17–2.29)	0.48
	Total (23,248)	1.05 (0.45–2.4)	0.9	Total (23,188)	1.01 (0.41–2.52)	0.96
Energy from EPA (%)	Female (14,729)	1.38 (0.15–12.3)	0.76	Female (14,690)	1.86 (0.17–19.53)	0.60
	Male (8,519)	6.42 (0.36–111.48)	0.2	Male (8,498)	0.3 (0.01–6.52)	0.44
	Total (23,248)	1.54 (0.27–8.7)	0.62	Total (23,188)	0.81 (0.12–5.25)	0.83

The crude and adjusted Cox proportional hazard model was used to calculate the hazard ratio and 95% confidence intervals. *Significant *p*-values (<0.05) are indicated with asterisk. a: Adjusted model a b: Adjusted model b.

After adjusting the menopause status in models, a and b, no association was found between n-3 FA and EPA as proportion of total energy (%), and absolute n-3 FA and EPA intake (g/d) (Supplementary Table 5). Higher HEI score was associated with reduced CVD incidence [OR: 0.97 (95%CI 0.90–0.98), *p*-v < 0.001, and HR: 0.98 (95% CI 0.97–0.98), *p*-v < 0.00]. Supplementary Table 4 shows the comparison of baseline characteristics between pre- and postmenopausal females.

### 3.4 The association of n-3 FA intake and HEI score with lipid biomarkers

Table 6 shows the adjusted linear regression model to assess the association of HEI score and n-3 FA intake with lipid biomarkers (RC, TG, LDL-C, non-HDL-C, and HDL-C). No association was found between n-3 FA intake (g/d) and lipid biomarkers except for HDL-C in the total cohort and females [coef: 0.033 (95%CI 0.009–0.057), *p*-v = 0.007 and coef: 0.038 (95%CI 0.006–0.071), *p*-v = 0.02, respectively]. N-3 FA as proportion of total energy (%) was associated with only HDL-C in the total cohort [coef: 0.08 (95%CI), *p*-v = 0.002]. In women, a higher HEI score was associated with lower RC, TG, non-HDL-C, and higher HDL-C [coef: −0.006 (95%CI), −0.01 (95%CI), −0.008 (95%CI), +0.004 (95%CI), *p*-v < 0.001], but no association was found with LDL-C. The same trend was found in males between HEI score and RC, TG, non-HDL-C, and HDL-C [coef: −0.01 (95%CI −0.018–−0.005), *p*-v < 0.001, coef −0.01 (95%CI −0.02–−0.006), *p*-v < 0.001, coef −0.008 (95%CI −0.014–−0.001), *p*-v = 0.01, coef: +0.005 (95%CI 0.003–0.007), *p*-v < 0.001]. In the total cohort, higher HEI score was associated with lower RC, TG, non-HDL-C and higher LDL-C and HDL-C [coef: −0.01 (95%CI −0.016–−0.011), *p*-v < 0.001, coef −0.02 (95%CI −0.02–−0.01), *p*-v < 0.001, coef −0.009 (95%CI −0.012–−0.005), *p*-v < 0.001, coef: +0.004 (95%CI 0.001–0.008), *p*-v = 0.003, coef: +0.014 (95%CI 0.012–0.015), *p*-v < 0.001 respectively].

**Table 6:** Linear regression for assessing the relation of dietary intakes with blood lipid profile.

Variable		RC (mmol/L)		TG (mmol/L)		LDL-C (mmol/L)		Non-HDL-C (mmol/L)		HDL-C (mmol/L)	
		Coefficient (95% CI)	*p*-v	Coefficient (95% CI)	*p*-v	Coefficient (95% CI)	*p*-v	Coefficient (95% CI)	*p*-v	Coefficient (95% CI)	*p*-v
N-3 FA intake(g/d)^a^	Female (5,709)	−0.03 (−0.08–0.02)	0.3	−0.05 (−0.13–0.02)	0.1	−0.006 (−0.08–0.06)	0.8	−0.03 (−0.11–0.04)	0.3	0.038 (0.006–0.071)	0.02*
	Male (3,038)	0.01 (−0.07–0.1)	0.7	0.007 (−0.11–0.13)	0.9	0.05 (−0.03–0.15)	0.2	0.07 (−0.01–0.16)	0.1	0.012 (−0.01–0.04)	0.4
	Total (8,747)	−0.01 (−0.06–0.03)	0.6	−0.02 (−0.09–0.04)	0.5	0.03 (−0.02–0.09)	0.2	0.02 (−0.03–0.07)	0.4	0.033 (0.009–0.057)	0.007*
Energy from N-3 FA (%)^a^	Female (5,709)	−0.06 (−0.16–0.31)	0.2	−0.09 (−0.24–0.06)	0.2	−0.004 (−0.14–0.14)	0.9	−0.07 (−0.21–0.07)	0.3	0.05 (−0.01–0.11)	0.1
	Male (3,038)	0.07 (−0.16–0.31)	0.5	0.1 (−0.21–0.42)	0.5	0.12 (−0.12–0.36)	0.3	0.19 (−0.03–0.42)	0.09	−0.001 (−0.07–0.07)	0.9
	Total (8,747)	−0.03 (−0.14–0.06)	0.5	−0.05 (−0.2–0.09)	0.4	0.08 (−0.03–0.21)	0.1	0.05 (−0.07–0.17)	0.4	0.08 (0.03–0.13)	0.002*
HEI score^b^	Female (5,709)	−0.006 (−0.009–−0.003)	<0.001*	−0.01 (−0.015–−0.006)	<0.001*	−0.002 (−0.006-0.002)	0.3	−0.008 (−0.012–−0.004)	<0.001*	0.004 (0.003–0.006)	<0.001*
	Male (3,038)	−0.01 (−0.018–−0.005)	<0.001*	−0.01 (−0.02–−0.006)	<0.001*	0.004 (−0.002-0.01)	0.2	−0.008 (−0.014–−0.001)	0.01*	0.005 (0.003–0.007)	<0.001*
	Total (8,747)	−0.01 (−0.016–−0.011)	<0.001*	−0.02 (−0.02–−0.01)	<0.001*	0.004 (0.001–0.008)	0.003*	−0.009 (−0.012–−0.005)	<0.00*1	0.014 (0.012–0.015)	<0.001*
EPA intake (g/d)^a^	Female (5,709)	−1.03 (−1.88–−0.26)	0.009*	−1.13 (−2.2–−0.04)	0.04*	1.24 (0.21–2.27)	0.01*	0.21 (−0.83–1.27)	0.6	0.35 (−0.09–0.79)	0.1
	Male (3,038)	−0.2 (−1.36–0.96)	0.7	0.47 (−1.06–2)	0.5	1.97 (0.79–3.15)	0.01*	1.77 (0.65–2.89)	0.002*	−0.03 (−0.41–0.33)	0.8
	Total (8,747)	−0.49 (−1.15–0.15)	0.1	−0.12 (−1.02–0.77)	0.7	1.4 (0.62–2.17)	<0.001*	0.9 (0.13-1.67)	0.02*	−0.09 (−0.41-0.22)	0.5
Energy from EPA (%)^a^	Female (5,709)	−1.62 (−2.96–−0.28)	0.01*	−1.85 (−3.74–0.03)	0.055	1.72 (−0.05–3.51)	0.058	0.11 (−1.7–1.9)	0.9	0.57 (−0.19–1.34)	0.1
	Male (3,038)	−0.21 (−2.98–2.56)	0.9	1.56 (−2.08–5.22)	0.4	4.5 (1.72–7.33)	0.002*	4.29 (1.62–6.97)	0.002*	−0.11 (−1.01-0.78)	0.8
	Total (8,747)	−1.01 (−2.31–0.28)	0.1	−0.47 (−2.25–1.29)	0.6	2.57 (1.04–4.09)	0.001*	1.55 (0.03-3.07)	0.04*	0.12 (−0.5-0.75)	0.7

The adjusted linear regression was used to calculate the coefficient (β) and 95% confidence intervals. *Significant *p*-values (<0.05) are indicated with asterisk. a: Adjusted model a. b: Adjusted model b.

## 4 Discussion

In our ATP cohort, we found that a higher HEI score was associated with lower risk and odds of CVD incidence. Also, a higher HEI score was associated with lower levels of RC, TG, non-HDL-C, and higher HDL-C in women, men, and the total cohort. Interestingly, no association was found between n-3 FA intake (g/d) and CVD incidence risk. However, higher energy from n-3 FA (%) increased the risk of CVD incidence in males but not in women. No association was found between n-3 FA intake (g/d) and energy from n-3 FA (%) with lipid biomarkers (TG, RC, LDL-C, and non-HDL-C). A higher intake of n-3 FA (g/d) was associated with higher HDL-C levels in females and the total cohort, but energy from n-3 FA (%) was associated with higher HDL-C only in the total cohort.

### 4.1 HEI score

Our results are generally consistent with previous observational studies that reported a higher HEI score is associated with lower CVD incidence (38–42). These studies mainly compared the highest vs. the lowest quartile of HEI score and found that higher quartiles have lower CVD incidence than the lowest quartile. In our study, we found that both females and males had lower CVD incidence in the moderate (scores 51–80) vs. low HEI (scores ≤50) category (24.5% vs. 21 and 29.5% vs. 27%, respectively). Also, we found that with every one unit HEI score increase, the odds and risk of having CVD in the future decreased by 3%.

Studies on HEI score and lipid biomarkers have mainly focused on the relation with TG, TC, LDL-C, and HDL-C. To the best of our knowledge, this is the first study assessing the association of HEI scores with non-fasting RC. The results of previous studies on lipid biomarkers are controversial. While some studies have reported a significant inverse association between HEI score and TG, LDL-C, and TC and a direct association with HDL-C (43–45), others have found no association between HEI score and lipid biomarkers (46–48). In this study, one unit increase in HEI score was associated with reduced levels of non-fasting RC, TG, and non-HDL-C (−0.01, −0.02, and −0.0009 (mmol/L), respectively); as well as increased levels of HDL-C (+0.014 (mmol/L), respectively) in both females and males.

In this study, higher HEI scores were associated with lower CVD incidence risk and lipid biomarkers. Thus, for prevention of CVD, a dietary pattern with a higher intake of total fruits and vegetables, whole fruits, dark green and orange vegetables, legumes, total and whole grains, milk, meats, beans, and non-hydrogenated vegetable oil (or oil in fish, nuts, and seeds), and moderate intake of saturated fats, sodium, sugar, and alcohol (34), is recommended. The Canadian Cardiovascular Society, the American College of Cardiology/American Heart Association, and the European Society of Cardiology guidelines recommend this dietary pattern for preventing and managing CVD (16, 17, 49). This dietary pattern is associated with higher plant-based foods, fiber, vitamins including vitamin C and niacin, minerals, beneficial unsaturated fats intake, and lower saturated and trans FA intake (49). From a mechanistic point of view, higher intake of fiber, vitamin C, and niacin, can reduce CVD incidence by lowering TC, LDL-C, and inflammatory markers (50–52). In the intestine, fiber binds to bile acids and reduces cholesterol absorption. This leads to a lower hepatic cholesterol pool. As a compensatory response, the liver upregulates LDL receptor expression to increase uptake of circulating LDL-C, resulting in reduced TC, LDL-C, and CVD risk (53). Vitamin C is also associated with reduced LDL-C by promoting the conversion of cholesterol to bile acids, thereby depleting the liver cholesterol pool. This initiates a similar compensatory mechanism, leading to reduced circulating TC and LDL-C. Additionally, vitamin C acts as an antioxidant that prevents atherosclerosis by inhibiting LDL oxidation (54, 55). Niacin reduces CVD risk by decreasing inflammation, TG, and LDL-C, while increasing HDL-C by inhibiting the catabolism of HDL apolipoprotein A-I (ApoA-I). Niacin also decreases fatty acid mobilization from adipose tissue and inhibits hepatocyte diacylglycerol acyltransferase-2, a key enzyme in triglyceride synthesis, thereby reducing the secretion of VLDL and LDL lipoproteins (56, 57). Saturated and trans FAs activate the sterol regulatory element binding protein (SREBP)-2 pathway which upregulates hepatic cholesterol synthesis and increases the LDL-C levels (58, 59). Therefore, lower intakes of saturated and trans FAs are associated with reduced risk of CVD (60). In this study, dietary intake of niacin and vitamin C increased progressively across low, moderate, and high HEI score categories (Supplementary Table 1).

### 4.2 N-3 fatty acid intake

The absolute intake of n-3 FA (g/d) for females was lower than males (1.3 vs. 1.6 g/d) but the n-3 FA as proportion of total energy (%) was marginally higher (0.7 vs. 0.6%) due to lower total energy intake in females. However, this difference in proportion of energy from n-3 FA for both females and males is minimal and not clinically significant. In our cohort, we observed a direct association between total n-3 FA intake and CVD incidence in males, but no association was found either in females or the total cohort.

#### 4.2.1 The cardioprotective effect of n-3 FA

N-3 FA cardioprotective effects are related to their role in reducing inflammation, increasing anti-inflammatory markers, reducing the expression of adhesion molecules, and lowering the TG levels (61, 62). Marine n-3 FA, especially EPA, is involved in these cardioprotective mechanisms. EPA and DHA are precursors for producing anti-inflammatory resolvins (63). EPA and DHA intake is associated with lower inflammatory markers by reducing the production of inflammatory eicosanoids, and cytokines (64, 65). Nuclear factor-kappaB (NF-κB) is a transcription factor that increases the expression of pro-inflammatory genes, including Tumor necrosis factor alpha (TNF-alpha). EPA has been shown to inactivate NF-κB (66). EPA decreases TG levels by increasing fatty acid oxidation and downregulating SREBP, a transcriptional factor that regulates cholesterol, TG, and fatty acid synthesis (67, 68).

Some observational studies reported an inverse association between n-3 FA intake (or fish) and CVD incidence or mortality (69–71), but some studies reported no association (72–74). The results of RCT studies are also controversial. REDUCE-IT trial study showed that 4 g/d Icosapent Ethyl (a purified n-3 EPA) resulted in a 25% decrease in CVD incidence, 18.3% decrease in TG, and 3.1% increase in LDL-C (27). However, the STRENGTH study (using 4 g/d carboxylic acid formulation of EPA and DHA) failed to improve CVD incidence but reduced TG (−19%) while increasing LDL-C (1.2%) (75). The controversy may be related to the health background of participants, choice of placebo, dosage, bioavailability, and source of the n-3 FA (76). Most of the studies that showed beneficial effect of n-3 FA on lipid biomarkers and CVD, used high dosage (>1 g/d) of marine n-3 FA (especially EPA) (76–78). For example, REDUCE-IT trial used purified EPA while STRENGTH trial used a combination of EPA and DHA. The level of EPA in participants’ serum in the REDUCE-IT trial was higher than STRENGTH, indicating the importance of bioavailability and EPA serum levels for beneficial effects (79).

#### 4.2.2 The clinical implications

Our results indicate a sex-specific effect of n-3 FA, which has been seen previously in other studies (79, 80). For example, Allaire J et al. observed that both EPA and DHA supplementation increase LDL-C in males more than females (79). Our results on lipid biomarkers are in line with previous studies that showed higher EPA and DHA intake are associated with reduced TG and RC while increasing LDL-C (27, 79, 80). However, the direct association of n-3 FA with CVD incidence in males was unexpected. This observation may be attributed to a number of factors. For example, the dosage and source of n-3 FA intake; studies have shown a dose-dependent association between marine n-3 FA intake (EPA and DHA) and decreased CVD incidence, TG, LDL-C, and increased HDL-C (77, 78). Most studies have shown that the minimum dosage for the cardioprotective effect of n-3 FA is 1 g/d of marine EPA and DHA, and the optimal is 3–4 g/d (77, 78). However, in our study, the mean intake of n-3 FA, EPA, and EPA + DHA was 1.4 g/d, 26 mg/d, and 80 mg/d, which are lower than the minimum dosage indicated in previous studies for cardioprotective effects. Mechanistic studies indicated that EPA, and not ALA, lowers inflammation and CVD risk by inactivating NF-κB expression. In our study, the n-3 FA intake source was mostly from ALA which may explain our observations (81).

The difference in physiology of CVD in males vs. females; males have a higher CVD risk than females (82), and n-3 FA has a sex-specific difference in effect (79).

The background diet of participants; out of 23,248 participants, 4 (0.02%) had a high HEI score (81–100). Previous studies in Canada showed that the prevalent dietary pattern in Canadians is a western diet with high intake of ultra-processed foods, sugar, and low intake of fruits, and vegetables, and this dietary pattern is associated with lower HEI score (34, 83, 84). Less than 1% of Canadian population older than 2 years had HEI score>80 in previous studies (34). Therefore, from a mechanistic standpoint, the intake of 1.6 g/d total n-3 FA in males may not be enough to modify the CVD risk while having a low-medium quality diet.

The association of n-3 FA with lipid biomarkers; in this study higher n-3 FA (g/d) intake was not associated with levels of LDL-C in males and females, but higher n-3 FA as proportion of energy (%) was associated with higher HDL-C in females. This suggests that n-3 FA might affect lipid biomarkers in females and males differently. However, more studies in future are needed to validate the sex-difference for the effect of n-3 FA on lipid biomarkers and CVD incidence and propose mechanisms for how n-3 FA is differently associated with CVD risk and lipid biomarkers in males vs. females.

### 4.3 Strengths and limitations

The dietary intake was measured only once at the beginning of the study and was not repeated during the study. Since individuals may alter their diet in the long term, we could not assess more recent dietary intake. Although dietary intake was assessed using a 12-month food frequency questionnaire, which may be subject to recall bias, the data were collected prospectively and not specifically focused on n-3 fatty acid intake. This prospective design, combined with subsequent ascertainment of CVD outcomes, strengthens the validity of the observed associations. Another key strength of this study is assessment of n-3 fatty acid intake in the context of overall diet quality, allowing for a more comprehensive evaluation of their combined impact on lipid biomarkers and CVD risk. This study provides understanding of how dietary patterns as a whole—rather than a single nutrient—may influence CVD risk. Also, this study is the first study that assessed the relation of diet quality and n-3 FA intake with non-fasting RC level. We recommend future studies assess the association of HEI scores using the newer version of the HEI score (2019). Also, we recommend RCT studies to assess the association of n-3 FA with CVD risk using a higher dosage (> 1 g/d of EPA + DHA).

## 5 Conclusion

Higher diet quality (as assessed using the HEI score) was associated with lower non-fasting TG and RC, and CVD incidence. Total N-3 FA intake (comprised largely of ALA) was not associated with CVD risk and lipid biomarkers, but higher EPA intake was associated with lower non-fasting RC and TG. The findings of this study reinforce the importance of maintaining a high-quality diet with a higher intake of plant-based foods, whole grains, and proteins for optimal health, including CVD risk.

## Data Availability

The datasets presented in this article are not readily available due to the confidentiality reasons and the privacy of identifiable information. Requests to access the datasets should be directed to deurich@ualberta.ca and proctor@ualberta.ca.

## Author disclaimer

This study is based in part on data provided by Alberta Health. The interpretation and conclusions contained herein are those of the researchers and do not necessarily represent the views of the Government of Alberta. Neither the Government nor Alberta Health expresses any opinion in relation to this study. ATP is only possible due to the commitment of its research participants, its staff, and its funders: Alberta Health, Alberta Cancer Foundation, Canadian Partnership Against Cancer, and Health Canada, and substantial in-kind funding from Alberta Health Services. The views expressed herein represent the views of the authors and not of ATP or any of its funders.

## Glossary

AH: Alberta health
ALA: Alpha linolenic acid
ATP: Alberta’s Tomorrow Project
BMI: Body mass index
CanPath: Canadian Partnership for Tomorrow’s Health
CDHQ: Canadian Diet History Questionnaire
CI: Confidence interval
Coef: Coefficient
CORE: Clinical outcomes in routine evaluation
CVD: Cardiovascular disease
DHA: Docosahexaenoic acid
DPA: Docosapentaenoic acid
EPA: Eicosapentaenoic acid
FA: Fatty acid
HDL-C: High-density lipoprotein cholesterol
HEI: Healthy Eating Index
HLQ: Health and Lifestyle Questionnaire
HR: Hazard ratio
IDL: Intermediate-density lipoproteins
LDL-C: Low-density lipoprotein cholesterol
MUFA: Mono-unsaturated fatty acid
NF-κB: Nuclear factor-kappaB
OR: Odds ratio
PUFA: Poly-unsaturated fatty acid
*p*-v: *p*-value
PYTPAQ: Past Year Total Physical Activity Questionnaire
RC: Remnant cholesterol
RCT: Randomized controlled trials
SD: Standard deviation
SFA: Saturated fatty acid
SREBP: Sterol regulatory element-binding proteins
TNF-alpha: Tumor necrosis factor alpha
VLDL: Very low-density lipoproteins
