# Supplementary material for: The association of healthy eating index score and n-3 fatty acid intake with cardiovascular diseases incidence and lipid biomarkers in Alberta’s tomorrow project cohort
Source: Front Nutr. 2025 Sep 24;12:1630126. doi: 10.3389/fnut.2025.1630126 (PMC12505668; doi:10.3389/fnut.2025.1630126)
Supplement: Supplementary file 1 [file Data_Sheet_1.pdf]

Table-1 The level of micronutrients across HEI score categories

| Variable | Low HEI score N=7,920 | Moderate HEI score N=15,324 | High HEI score N=4 | P-V for Low vs moderate HEI score | P-V for moderate vs high HEI score |
|---|---|---|---|---|---|
| **Niacin (mg)** | 17.30±8.46 | 22.36±10.03 | 37.75±20.90 | **<0.001** | **0.02** |
| **Vitamin C (mg)** | 85.82±54.18 | 175.38±108.01 | 463.80±396.63 | **<0.001** | **0.03** |
| **SFA (g)** | 21.93±12.20 | 22.92±13.01 | 14.53±2.79 | **<0.001** | 0.09 |

Abbreviations: saturated fatty acid (SFA)

HEI score categories are defined as follows: 0-50: low, 51-80: moderate, 81-100: high.

Variables are reported as mean±SD. Significant p-values (<0.05) are shown in bold. An independent two sample t-test was used to compare the means between the low and moderate HEI score groups. A two-sample Wilcoxon rank-sum (Mann-Whitney) test was used to compare the means between the moderate and high HEI score.

For the independent two-sample t-test, the normality of variables was assessed. Vitamin C, SFA, and niacin did not follow a normal distribution, so an unequal variances t-test was applied. For the two-sample Wilcoxon rank-sum (Mann-Whitney) test, no assumption of normality is required.

Table-2 Mean SBP and DBP stratified by sex and CVD incidence, and their association with HEI score

| | | **SBP (mmHg)** | | **DBP (mmHg)** | |
|---|---|---|---|---|---|
| **Groups** | | mean±SD | P-V | mean±SD | P-V |
| **Female with CVD (n=1,186)** | | 125.93±15.70 | **<0.001** | 73.77±10.14 | 0.5 |
| **Females without CVD (4,424)** | | 121.32±15.09 | | 73.58±9.8 | |
| **Males with CVD (n=911)** | | 131.96±14.64 | **<0.001** | 76.57±10.24 | 0.1 |
| **Males without CVD (n=2,229)** | | 129.93±13.56 | | 77.20±9.62 | |
| **Variable** | | **Coefficient (95%CI)** | | **Coefficient (95%CI)** | |
| **HEI score** | Female (n=5,610) | -0.07 (-0.14- -0.00) | **0.03** | -0.05 (-0.09- -0.01) | **0.01** |
| | Male (3,140) | -0.06 (-0.15- 0.02) | 0.14 | -0.03 (-0.09- 0.03) | 0.3 |

The total number of participants with available blood pressure data was n = 8,750 (64.1% females and 35.9% males).

Abbreviations: systolic blood pressure (SBP), diastolic blood pressure (DBP), cardiovascular diseases (CVD), healthy eating index (HEI), standard deviation (SD), confidence interval (CI), odds ratio (OR).

Continuous variables are reported as mean ± SD. Significant p-values (<0.05) are shown in bold.

SBP and DBP for females with versus without CVD were normally distributed; the mean difference was assessed using an equal-variance two-sample independent t-test.

SBP and DBP for males with versus without CVD were not normally distributed; the mean difference was assessed using an unequal-variance two-sample independent t-test.

The association of HEI score with SBP and DBP was assessed using adjusted linear regression (coefficients and 95% confidence intervals are reported). Age, BMI (kg/m2), total energy (kcal), carbohydrate (%), protein (%), and fat (%) intake, Elixhauser index, and n-3 fatty acid intake (g/d) were adjusted.

Table-3 Logistic regression assessing the association of SBP and DBP with CVD incidence (Odds Ratios)

| **Variables** | | **OR (95%CI)** | **P-V** |
|---|---|---|---|
| **SBP (mmHg)** | Female (n=5,610) | 1.01 (1.00-1.01) | **<0.001** |
| | Male (3,140) | 1.00 (0.99-1.00) | 0.22 |
| **DBP (mmHg)** | Female (n=5,610) | 0.99 (0.99-1.00) | 0.85 |
| | Male (3,140) | 0.99 (0.98-1.00) | 0.36 |

The total number of participants with available blood pressure data was n=8,750 (64.1% females and 35.9% males).

Abbreviations: systolic blood pressure (SBP), diastolic blood pressure (DBP), confidence interval (CI), odds ratio (OR).

The association of SBP and DBP with CVD incidence was assessed using adjusted logistic regression (odds ratios and 95% confidence intervals are reported). Age, BMI (kg/m2), total energy (kcal), carbohydrate (%), protein (%), and fat (%) intake, Elixhauser index, n-3 fatty acid intake (g/d), and HEI score were adjusted. Significant p-values (<0.05) are in bold.

Out of 14,729 females, menopause data were missing for n=4,432. A subset of females (n=10,297) with available data on menopause status was included in the analysis. Among these females, 30.65% were premenopausal, while 69.35% were postmenopausal.

Table-4 Baseline characteristics of females stratified by menopause status

| Variables | Pre-menopause (N=3,156) | Post-menopause (N=7,141) | p-v |
|---|---|---|---|
| **Age at baseline (years)** | 50.47±9.63 | 54.22±8.51 | **<0.001** |
| **CVD incidence (number (%))** | 720 (22.81%) | 1,890 (26.47%) | **<0.001** |
| **BMI (kg/m$^2$)** | 28.70±6.40 | 28.64±6.25 | 0.6 |
| **Total Energy intake (kcal)** | 1628.16±650.50 | 1622.90±650.03 | 0.7 |
| **Energy from carbohydrates (%)** | 51.27±8.56 | 51.81±8.39 | **0.002** |
| **Energy from fat (%)** | 32.67±6.99 | 32.36±6.91 | **0.04** |
| **Energy from protein (%)** | 16.24±2.93 | 15.99±2.92 | **<0.001** |
| **Energy from PUFA (%)** | 7.27±2.16 | 7.27±2.11 | 0.9 |
| **Energy from SFA (%)** | 10.63±2.83 | 10.48±2.82 | **0.01** |
| **Energy from MUFA (%)** | 12.18±2.94 | 12.06±2.90 | **0.04** |
| **Energy from n-3 FA (%)** | 0.73±0.22 | 0.73±0.22 | 0.05 |
| **N-3 FA intake (g/d)** | 1.31±0.63 | 1.33±0.66 | 0.1 |
| **EPA intake (g/d)** | 0.02±0.030 | 0.02±0.03 | 0.2 |
| **Energy from EPA (%)** | 0.01±0.01 | 0.01±0.01 | 0.05 |
| **HEI score** | 54.90±9.65 | 54.94±9.59 | 0.8 |
| **Physical activity level** | 1.90±0.39 | 1.88±0.40 | **0.004** |
| **Elixhauser score** | 2.40±1.97 | 1.85±1.77 | **<0.001** |
| | **Pre-menopause (N=1,217)** | **Post-menopause (N=3,116)** | |
| **SBP (mmHg)** | 122.15±15.07 | 124.38±15.56 | **<0.001** |
| **DBP (mmHg)** | 74.47±9.75 | 73.48±9.87 | **0.002** |

Abbreviations: cardiovascular diseases (CVD), body mass index (BMI), poly-unsaturated fatty acid (PUFA), saturated fatty acid (SFA), mono-unsaturated fatty acid (MUFA), fatty acid (FA), eicosapentaenoic acid (EPA), healthy eating index (HEI), systolic blood pressure (SBP), diastolic blood pressure (DBP).

The total number of females with available SBP and DBP data was n = 4,333 (72% postmenopausal and 28% premenopausal). Continuous variables are reported as mean ± SD, and categorical variables are reported as number (percentage). Significant p-values (<0.05) are shown in bold. CVD incidence was compared using Fisher's exact test. An independent two-sample t-test was used to compare means between groups.

Age, energy from carbohydrates, energy from fat, energy from protein, energy from MUFA, energy from SFA, Elixhauser score, physical activity level (PAL), DBP, and SBP followed a normal distribution; thus, mean differences were analyzed using an equal-variance two-sample independent t-test.

BMI, total energy intake, energy from PUFA, n-3 fatty acid intake, energy from n-3 fatty acids, energy from EPA, EPA intake, and HEI score did not follow a normal distribution; therefore, mean differences were analyzed using an unequal-variance two-sample independent t-test.

The energy contribution of macro- and micronutrients (%) was calculated as the mean energy from the specific macro-/micronutrient (kcal) divided by the mean total energy intake (kcal), multiplied by 100.

The age at menopause was missing for 3,951 (55%) of the 7,141 postmenopausal females. Therefore, to assess whether menopause is associated with future CVD incidence, these individuals were excluded from the analysis. In the resulting subset of 6,346 females, 3,190 (50.3%) were postmenopausal and 3,156 (49.7%) were premenopausal. Females with prevalent menopause (n=3,161) were defined as those who had undergone menopause before CVD incidence, while females without prevalent menopause (n=3,185) included those who experienced menopause after CVD incidence (n=29) or had not yet undergone menopause (n=3,156). Logistic regression was used to assess the association of menopause status with future CVD incidence. No significant association was found between menopause status and CVD incidence in future before and after adjusting for BMI ($kg/m^2$), total energy intake (kcal), energy from carbohydrate, fat, and protein (%), total n-3 FA intake (g/d), HEI score, and Elixhauser index [crude OR:1.06 (95%CI:0.95-1.19), p-v=0.26, adjusted OR:1.12 (95%CI: 0.99-1.26), p-v=0.05]. Table-5 shows the association of HEI score and N-3 FA intake with CVD incidence after adding menopause status to adjusted confounders in models a and b.

Table-5 Assessing the relation of dietary variables with odds and risk of CVD incidence

| Variable | OR (95%CI) | p-v | HR (95%CI) | p-v |
|---|---|---|---|---|
| **N-3 FA intake (g/d) [a]** | 1.10 (0.91-1.32) | 0.3 | 1.06 (0.91-1.24) | 0.4 |
| **Energy from N-3 FA (%) [a]** | 1.29 (0.90-1.89) | 0.1 | 1.18 (0.87-1.59) | 0.2 |
| **N-3/N-6 PUFA ratio [a]** | 1.04 (0.08-12.57) | 0.9 | 0.86 (0.11-6.69) | 0.8 |
| **EPA intake (g/d) [a]** | 0.93 (0.08-9.83) | 0.9 | 0.84 (0.11-6.30) | 0.8 |
| **Energy from EPA (%) [a]** | 2.27 (0.035-144.16) | 0.7 | 1.80 (0.05-57.65) | 0.7 |
| **HEI score [b]** | 0.97 (0.90-0.98) | **<0.001** | 0.98 (0.97-0.98) | **<0.001** |

Abbreviations: confidence interval (CI), cardiovascular diseases (CVD), odds ratio (OR), confidence interval (CI), hazard ratio (HR), poly-unsaturated fatty acid (PUFA), fatty acid (FA), eicosapentaenoic acid (EPA), healthy eating index (HEI).

Logistic regression was used to calculate the odds ratio and 95% confidence intervals. The Cox proportional hazard model was used to calculate the hazard ratio and 95% confidence intervals. Significant p-values (<0.05) are shown in bold.

a: Adjusted model a, and menopause prevalence

b: Adjusted model b, and menopause prevalence.
